# Non-parathyroid Hypercalcaemia in a Patient on Lithium Therapy: A Case Report

**DOI:** 10.7759/cureus.95945

**Published:** 2025-11-02

**Authors:** F M Tanvir Alam, Zarin Tasnim Parisa, Francis Donya, Anurag Sharma

**Affiliations:** 1 Internal Medicine, Medway NHS Foundation Trust, Kent, GBR; 2 Geriatrics, William Harvey Hospital in Ashford, Ashford, GBR

**Keywords:** bipolar affective disorder, hypercalcaemia, lithium-induced hypercalcemia, mood stabilizer, non-hyperparathyroid

## Abstract

Lithium is a commonly used drug in the treatment of several psychiatric disorders. However, due to its narrow therapeutic range, vigilant monitoring is essential to detect adverse effects. While hypercalcaemia is a documented complication of lithium therapy, it is typically associated with hyperparathyroidism. This case report presents a 67-year-old female on prolonged lithium therapy who developed refractory hypercalcaemia that was not attributable to hyperparathyroidism. Discontinuation of lithium resulted in normalisation of calcium levels, confirming lithium-induced hypercalcaemia in the context of normal parathyroid hormone (PTH) levels. The existing literature predominantly discusses hypercalcaemia as a consequence of hyperparathyroidism in lithium users. This case challenges that paradigm, suggesting an alternative, as yet undefined, mechanism for lithium-induced hypercalcaemia.

## Introduction

Lithium is a well-established mood stabiliser and has been a cornerstone in the treatment of bipolar disorder and other mood disorders. Its effectiveness as a mood stabiliser is supported by evidence that its long-term use reduces suicide risk and overall mortality in individuals with mood disorders [1]. Because of its narrow therapeutic range, long-term lithium use requires careful monitoring for adverse effects such as cognitive impairment, thyroid and parathyroid dysfunction, renal impairment, and electrolyte imbalances [2]. Numerous reports of hypercalcaemia have been documented in patients using lithium, and this has mainly been observed as a complication of lithium-induced hyperparathyroidism [3]. As a potentially life-threatening complication, a full understanding of the pathophysiology of hypercalcaemia associated with lithium use is important to better guide prevention, identification, and intervention.

There are currently two main proposed mechanisms of lithium-induced hypercalcaemia. The first is competitive inhibition by lithium of the calcium-sensing receptors (CSRs) found in the parathyroid glands. This would require higher serum calcium levels to trigger negative feedback on parathyroid hormone (PTH) secretion. Until this new threshold is reached, both serum PTH and serum calcium levels remain high [4,5]. The second theory describes a direct insult to the parathyroid glands by lithium, resulting in either a parathyroid adenoma or parathyroid gland hyperplasia, both causing raised serum PTH and subsequent hypercalcaemia [6]. Both mechanisms involve raised PTH.

Our aim in this case report is to present a rare phenomenon of lithium-induced hypercalcaemia with normal serum PTH levels.

## Case presentation

A 67-year-old female of South Asian background with a past medical history of bipolar disorder only, for which she had been receiving continuous lithium therapy (dose: 520 mg orally, twice daily) for more than 15 years, presented to the emergency department with an acute onset of non-epileptic seizures, which required management in the Intensive Care Unit.

She was later found to have hypercalcaemia ranging from 2.66 to 3.34 mmol/L (normal range, 2.20-2.60 mmol/L), validated by albumin levels between 31 and 38 g/L (normal range, 35-50 g/L). A review of her blood test records prior to admission revealed that the hypercalcaemia was pre-existing (see Table 1). The cause was not clearly identifiable. Initially, it was attributed to dehydration (see Table 2). However, the hypercalcaemia was resistant to optimal medical management, including aggressive intravenous rehydration with 0.9% normal saline, loop diuretics with furosemide, and intravenous bisphosphonate.

**Table 1:** Lithium levels and biochemistry

Variables	Serum Lithium (0.4-1 mmol/L)	Serum Adjusted Calcium (2.20-2.60 mmol/L)	Serum Albumin (35-50 g/L)	Serum Parathyroid Hormone (12-88 ng/L)
Before admission (months)				
18	0.66	2.79	42	-
9	0.68	2.63	40	-
2	-	2.90	40	-
1	0.61	2.72	33	-
Hospital admission				
Day 1	-	3.34	35	
Day 3	-	2.98	31	49
Day 5	0.44	2.85	32	
Day 7	0.61	2.71	29	
Day 15	0.57	2.66	37	
Day 22	-	2.82	38	
Lithium stopped				
Repeat level after 14 days	<0.04	2.47	35	
Repeat level after 21 days	-	2.51	34	

**Table 2:** Relevant biochemical findings, including renal functions and electrolytes eGFR: estimated glomerular filtration rate.

Variables	Serum Creatinine (59-104 µmol/L)	eGFR	Serum Sodium (133-146 mmol/L)	Serum Potassium (3.5-5.3 mmol/L)	Serum Urea (2.5-7.8 mmol/L)	24 Hour Urinary Calcium (2.5-7.5 mmol/24 hours)
9 months prior to admission	80	72	135	4.2	4.4	
During admission						
Day 1	91	64	144	4.5	8.9	
Day 3	87	66	140	4.0	6.7	3.8
Day 7	79	76	138	3.8	5.9	

Consequently, a workup for refractory hypercalcaemia was performed with input from a multidisciplinary team. Endocrine causes of hypercalcaemia, including hyperparathyroidism, thyrotoxicosis, adrenal insufficiency, and pheochromocytoma, were investigated and found to be normal. The patient's serum PTH was within the normal range at 49 ng/L (normal range, 12-88 ng/L). Thyroid function tests were also normal, with TSH 0.72 mIU/L (normal range, 0.30-4.80 mIU/L) and T4 17.4 pmol/L (normal range, 7.7-20.6 pmol/L).

The possibility of malignancy was also investigated. Evaluation for haematological malignancy, including myeloma screening, revealed no abnormalities. Full blood count, peripheral blood film, erythrocyte sedimentation rate (ESR), lactate dehydrogenase (LDH), calcium, parathyroid hormone (PTH), and vitamin D were all within normal parameters. Targeted imaging studies did not reveal any solid tumours classically associated with paraneoplastic hypercalcaemia, including those of pulmonary, renal, ovarian, and head and neck origin, nor was there evidence of lymphoma. Biochemical results further supported this exclusion, with parathyroid hormone-related protein (PTHrP) and calcitriol levels falling within normal reference ranges. CT chest-abdomen-pelvis was negative for any tumours or metastases. Tumour markers were all within the normal range (see Table 3). Altogether, endocrine, haematological, oncological, and other medical causes of hypercalcaemia were excluded.

**Table 3:** Tumour marker results during admission

Alpha-fetoprotein (kU/L) (Reference < 7.4)	CA 15-3 (kU/L) (Reference 0-23)	CEA (µg/L) (Reference: Non-smoker < 5, Smoker < 10)
3.5	11	2

A review of her medication history revealed no use of agents known to induce hypercalcaemia, including exogenous calcium or vitamin D preparations or any other supplements, either before admission or during hospitalisation. Lithium was the only long-term medication she was taking. With a suspicion of lithium-induced hypercalcaemia, lithium was discontinued, and calcium levels were monitored. After 14 days of follow-up, serum lithium of <0.04 mmol/L (normal range, 0.40-1.00 mmol/L) was recorded, confirming no residual bioavailability. At this point, serum calcium had normalised to 2.47 mmol/L (normal range, 2.20-2.60 mmol/L). At 21 days, serum calcium remained normal at 2.51 mmol/L (see Table 1).

Based on this information, this was deemed to be a case of lithium-induced hypercalcaemia not secondary to hyperparathyroidism.

## Discussion

The prolonged use of lithium is widely acknowledged as a risk factor for hypercalcaemia. Predominantly, existing literature cites hyperparathyroidism as an intermediary phenomenon. There are only two reports discussing non-parathyroid hypercalcaemia in patients receiving lithium therapy. Komatsu (1995) describes a case-control study in which 13 subjects on lithium therapy were found, on average, to have hypercalcaemia without biochemically significant hyperparathyroidism [7]. Dorflinger (2019) reports a case in which a patient on long-term lithium therapy experienced hypercalcaemia with a normal serum PTH and no other identifiable cause [8]. The findings of these reports deviate from the prevailing literature suggesting that hypercalcaemia in lithium use occurs only secondary to hyperparathyroidism. The significance lies in the implication of an unidentified mechanism. Therefore, hypercalcaemia as a complication of lithium therapy with a normal serum PTH remains an especially underreported phenomenon.

From a clinical standpoint, this case highlights the complexity of interpreting hypercalcaemia in lithium-treated patients. In routine practice, elevated calcium levels often trigger an endocrine workup that focuses on hyperparathyroidism. However, as demonstrated here, parathyroid-independent hypercalcaemia must also be considered. Persistent calcium elevation despite normal PTH levels, absence of malignancy, and lack of vitamin D supplementation should prompt clinicians to reassess lithium’s direct contribution. Early recognition can prevent unnecessary investigations and allow timely dose reduction or discontinuation before irreversible renal or skeletal complications occur.

The decision to withdraw lithium should always balance psychiatric stability against metabolic safety. Abrupt discontinuation can precipitate affective relapse; therefore, a multidisciplinary approach involving psychiatry, endocrinology, and general medicine is essential. Regular monitoring of serum calcium, renal function, and PTH during long-term therapy is vital. For patients requiring continuation of lithium due to psychiatric illness, adjunctive use of calcium-lowering agents or switching to alternative mood stabilisers, such as valproate, may be considered on a case-by-case basis.

The strength of this case lies in documenting a rare example of hypercalcaemia associated with lithium despite normal parathyroid function, suggesting an alternative pathway of calcium dysregulation. The comprehensive evaluation performed strengthens confidence in this association by excluding other recognised causes. However, as a single-patient observation, broader conclusions should be drawn with caution. Additional case reports or larger case series are required to determine how often lithium alone may contribute to hypercalcaemia independently of hyperparathyroidism and to clarify the underlying biological mechanisms.

## Conclusions

This case reinforces a clear temporal and causal relationship between chronic lithium exposure and the development of hypercalcaemia in the absence of parathyroid dysfunction. All recognised alternative aetiologies, including endocrine, malignant, nutritional, renal, and medication-related causes, were comprehensively excluded. Notably, the patient’s calcium concentration normalised only after lithium cessation, strongly supporting lithium as the sole driver of hypercalcaemia independent of the parathyroid axis.

Clinicians should recognise that lithium alone can provoke clinically significant hypercalcaemia, even when PTH levels are normal. Regular monitoring of serum calcium is essential in patients receiving long-term lithium therapy. Nevertheless, the risks and benefits of modifying lithium treatment must be carefully weighed for each patient presenting with hypercalcaemia. In this case, discontinuing lithium was appropriate; however, this decision may not be suitable for all patients.
